# Role of glycosylation-related gene MGAT1 in pancreatic ductal adenocarcinoma

**DOI:** 10.3389/fimmu.2024.1438935

**Published:** 2024-08-01

**Authors:** Lai Jiang, Jie Liu, Shengke Zhang, Chenglu Jiang, Jinbang Huang, Haiqing Chen, Xuancheng Zhou, Yiping Fu, Zhongqiu Yang, Rui Wang, Guanhu Yang, Hao Chi, Bo Li

**Affiliations:** ^1^ Clinical Medical College, Southwest Medical University, Luzhou, China; ^2^ Department of General Surgery (Hepatopancreatobiliary Surgery), The Affiliated Hospital of Southwest Medical University, Luzhou, Sichuan, China; ^3^ Department of General Surgery, Dazhou Central Hospital, Dazhou, China; ^4^ Department of Specialty Medicine, Ohio University, Athens, OH, United States; ^5^ Academician (Expert) Workstation of Sichuan Province, Metabolic Hepatobiliary and Pancreatic Diseases Key Laboratory of Luzhou City, The Affiliated Hospital of Southwest Medical University, Luzhou, Sichuan, China

**Keywords:** single-cell analysis, immunotherapy, predictive marker, glycosylation, glycosyltransferase, macrophages

## Abstract

**Background:**

pancreatic ductal adenocarcinoma (PDAC) is a malignant tumor with a very poor prognosis and a complex tumor microenvironment, which plays a key role in tumor progression and treatment resistance. Glycosylation plays an important role in processes such as cell signaling, immune response and protein stability.

**Materials and methods:**

single-cell RNA sequencing data and spatial transcriptome data were obtained from GSE197177 and GSE224411, respectively, and RNA-seq data and survival information were obtained from UCSC Xena and TCGA. Multiple transcriptomic data were comprehensively analyzed to explore the role of glycosylation processes in tumor progression, and functional experiments were performed to assess the effects of MGAT1 overexpression on PDAC cell proliferation and migration.

**Results:**

In PDAC tumor samples, the glycosylation level of macrophages was significantly higher than that of normal samples. MGAT1 was identified as a key glycosylation-related gene, and its high expression was associated with better patient prognosis. Overexpression of MGAT1 significantly inhibited the proliferation and migration of PDAC cells and affected intercellular interactions in the tumor microenvironment.

**Conclusion:**

MGAT1 plays an important role in PDAC by regulating glycosylation levels in macrophages, influencing tumor progression and improving prognosis.MGAT1 is a potential therapeutic target for PDAC and further studies are needed to develop targeted therapeutic strategies against MGAT1 to improve clinical outcomes.

## 1 Introduction

Pancreatic ductal adenocarcinoma (PDAC) is one of the most aggressive malignancies with a very poor prognosis. Despite advances in molecular and genetic research, the five-year survival rate for PDAC remains below 10% (1). Key characteristics of PDAC include late-stage diagnosis, rapid disease progression, and resistance to conventional therapies. The tumor microenvironment (TME) of PDAC is complex, comprising cancer cells, stromal cells, immune cells, and an extensive extracellular matrix (2–4). These components collectively promote tumor growth, metastasis, and therapeutic resistance. Understanding the interactions within this microenvironment is crucial for developing effective treatment strategies (5).

Glycosylation is a post-translational modification process in which glycans are enzymatically attached to proteins or lipids. This process plays a critical role in various biological processes, including cell signaling, immune response, and protein stability. Aberrant glycosylation is a hallmark of cancer, influencing tumor progression, metastasis, and immune evasion. Glycosyltransferases, the enzymes responsible for glycan synthesis and modification, are often dysregulated in tumors. This dysregulation can lead to the expression of unique glycan structures not present in normal tissues, affecting cell-cell interactions, signal transduction, and immune recognition (6). Therapeutic strategies targeting glycosylation pathways offer a novel approach to cancer treatment. Glycosyltransferase or glycosidase inhibitors, as well as monoclonal antibodies targeting specific glycan structures, are being explored to inhibit tumor growth and metastasis (7, 8).

In pancreatic cancer, research on glycosylation is gaining increasing attention, particularly the role of glycosyltransferases (9). These enzymes are involved in the biosynthesis of complex N-glycans, and their expression changes are closely related to alterations in cell adhesion, migration, and immune evasion. Understanding the impact of glycosylation on immune cells within the tumor microenvironment can reveal new biological mechanisms of tumor progression and identify new therapeutic targets (10).

## 2 Materials and methods

### 2.1 Source of raw data

The pancreatic cancer single-cell sequencing data in this study were obtained from the GSE197177 dataset in the Gene Expression Omnibus (GEO) database, and the spatial transcriptome data were obtained from the GSE224411 dataset. In addition, RNA-seq data for pancreatic cancer from the UCSC Xena platform (https://xena.ucsc.edu/) were downloaded from the TCGA (The Cancer Genome Atlas) cohort, which contains sequencing information for a total of 183 samples, and the corresponding survival data for survival analysis.

### 2.2 Processing of single-cell sequencing data

In this study, we conducted an in-depth analysis of PDAC single-cell RNA sequencing data using the Seurat package (version 4.3.0) (11). To ensure the accuracy of the analysis, strict quality control measures were implemented to filter the cells. Cells were screened based on the criteria that each cell must express between 200 and 4000 genes and have a mitochondrial gene expression percentage of less than 10%. Normalization was then performed using the NormalizeData function to eliminate the effects of sequencing depth. The FindVariableFeatures function was used to identify 2,000 highly variable genes, which were then subjected to Principal Component Analysis (PCA) and cell clustering. UMAP was used to visualize the clustering results. During cell type identification, we referenced cell marker gene information from pancreatic tissues in the CellMarker database (http://xteam.xbio.top/CellMarker/index.jsp) to ensure accurate cell type identification.

To explore metabolic state differences between normal and tumor tissues, we used the scMetabolism package (version 0.2.1) to quantitatively analyze metabolic pathway activities in single-cell data. This package includes human metabolic gene sets covering 85 KEGG pathways and 82 REACTOME entries, utilizing the VISION algorithm to score each cell for activity levels in these pathways. This method allowed us to quantify metabolic pathway activities at the cellular level, providing a powerful tool for understanding how cells regulate their metabolic states under different physiological and pathological conditions.

We used five algorithms, AddModuleScore, ssGSEA, AUCell, UCell, and singscore, to perform gene set scoring on single-cell data based on 185 glycosyltransferase-related genes (12, 13). This multi-method approach provides robust scoring, reduces errors and biases in the gene set scoring process, and provides comprehensive, robust and biologically meaningful insights. By means of scoring, we quantified the levels of glycosylation in cells of various cell types in both tumor and normal groups.

To comprehensively analyze cell communication patterns, we used the CellChat package (version 1.6.1) (14). CellChat modelled and analyzed intercellular communication by considering gene expression data and known interactions between signal transduction ligands, receptors and cofactors. Macrophages were divided into two groups based on glycosylation scores, and differences between high and low level glycosylated macrophages were compared by cell communication analysis. In addition to CellChat, we also utilize the CellCall software package (version 1.0.7) (15). The CellCall package integrates intracellular and intercellular signals to infer communication networks and internal regulatory signals that form the L-R-TF axis, and includes pathway activity analyses to assess changes in receptor cellular pathways induced by intercellular communication.

For enrichment analysis of different cell types in PDAC single-cell transcriptome data, particularly macrophages, we used the clusterProfiler (version 4.6.2) and GSVA (version 1.50.1) packages (16, 17). The clusterProfiler package supports querying multiple biological information databases, including GO, KEGG, and Reactome, while GSVA efficiently handles large datasets, allowing precise evaluation of gene set enrichment at the single-cell level.

Finally, the ggplot2 package (version 3.4.2) was the core tool for visualizing our results, offering a powerful and flexible way to create complex graphics based on the grammar of graphics.

### 2.3 Processing of spatial transcriptome sequencing data

Spatial transcriptomics data were processed and analyzed using the Seurat package (version 4.3.0). We applied the “SCTransform” function to normalize and scale the UMI counts to identify the most variable features. The “RunPCA” function was then used to perform dimensionality reduction to simplify the data structure. The “SpatialFeaturePlot” function was used for visualization after dimensionality reduction and clustering, allowing us to display cell subpopulation distributions in the spatial context of tissue slices, thus providing deeper insights into the spatial organization and functional status of cells.

The scMetabolism package was also applied to the spatial transcriptomics data. By evaluating the metabolic characteristics of different cell clusters in the dimensionally reduced and clustered spatial transcriptomics data, we could uncover important metabolic differences. This approach is invaluable for understanding the functional heterogeneity of cells and their interactions, thereby enhancing our comprehension of tumor complexity from both spatial and functional perspectives.

Using the Monocle package, we conducted pseudotime analysis to reveal the developmental and differentiation processes of cell clusters located at different positions within the spatial transcriptomics data. To run stlearn in Python, we employed the Scanpy package to perform preprocessing, visualization, clustering, pseudotime analysis, and differential expression analysis on the spatial transcriptomics data.

### 2.4 Spatial transcriptomics data combined with single-cell sequencing for deconvolution analysis

We used deconvolution analysis to infer the proportions of different cell types from mixed cell samples. This method combines the advantages of single-cell sequencing, which provides cell-level gene expression information within tissues, and spatial transcriptomics, which offers spatial location information of cells within tissues, revealing spatial heterogeneity. This approach enables a deeper understanding of tumor complexity and heterogeneity at high spatial resolution.

During the deconvolution analysis, we implemented RCTD (Robust Cell Type Decomposition) using the spacexr package (version 2.2.1). First, we constructed a reference model using the Reference function, based on annotated single-cell transcriptome data, providing essential baseline information for subsequent analysis. Next, we loaded the spatial transcriptomics data using the SpatialRNA function, forming a SpatialRNA object, a crucial step ensuring the accurate integration of spatial information. We then created an RCTD object using the create. RCTD function and estimated the relative proportions of each cell type within the mixed cell population by estimating the specific gene expression patterns of each cell type and using the least-squares method. This analysis not only provided proportion estimates of each cell type within the mixed samples but also inferred the proportion distribution of different cell types within each spot in the spatial transcriptomics data. Combining single-cell and spatial transcriptomics data in the deconvolution analysis allowed our study to reveal the cellular composition and spatial heterogeneity of tumor tissues at an unprecedented level of detail.

To further understand the cell-cell communication patterns within the tumor microenvironment, we used the mistyR package (version 1.6.1) for spatial transcriptomics data interaction analysis. mistyR is a powerful package specifically designed for spatial transcriptomics data analysis, capable of utilizing spatial location and gene expression data to effectively uncover interactions between cells within tissues. By calculating the spatial proximity between cells, mistyR can infer potential cell-cell communication networks, offering new insights into how cells interact spatially within the tumor microenvironment.

### 2.5 Prognostic analysis of MGAT1 gene expression in macrophages using bulk data

We explored the potential clinical prognostic value of MGAT1 expression in macrophages. To this end, we conducted an in-depth analysis combining bulk sequencing data with single-cell data. Using the Seurat package to process single-cell sequencing data, we first categorized macrophages from patient tumor tissues into MGAT1-positive and MGAT1-negative groups based on their MGAT1 gene expression. Next, we identified marker genes for these two subgroups using the FindAllMarkers function.

After identifying the marker genes for MGAT1-positive macrophages, we quantified these genes in bulk sequencing data to construct high-risk and low-risk groups. To evaluate the expression of these cell subgroups in invasive tumors, we processed the bulk RNA-seq data and performed single-sample gene set enrichment analysis (ssGSEA) using the GSVA method. This approach allowed us to quantitatively assess the enrichment levels of these marker gene sets in various tumor samples.

Finally, we combined these enrichment analysis results with clinical survival data to evaluate the clinical prognostic differences between the different risk groups. For survival analysis, we used the survminer (version 0.4.9) and survival (version 3.4-0) packages. Initially, we determined the optimal risk group cut-off point using the surv_cutpoint function. Based on this threshold, samples were divided into high-risk and low-risk groups, and survival curves were constructed using the survfit function.

Through this series of analyses, we not only revealed the potential role of MGAT1-positive macrophages in tumor prognosis but also provided important biomarkers for future clinical applications.

### 2.6 Cell culture and transient transfection

In our experimental study, we used two pancreatic cancer cell lines, SW1990 and PANC-1. These cell lines were obtained from the cell bank of the Central Laboratory at the Affiliated Hospital of Southwest Medical University. To ensure normal growth and maintenance of these cells, we cultured them in DMEM (HyClone) medium supplemented with 10% fetal bovine serum (HyClone), 100 U/L penicillin, and 100 mg/L streptomycin (Thermo Fisher Scientific). Standard culture conditions were maintained, including a 5% carbon dioxide environment, to ensure optimal cell viability and experimental consistency.

For transient transfection experiments, we used Lipofectamine 3000 (Invitrogen, Carlsbad, CA, United States) as the transfection reagent. Following the manufacturer’s instructions, we transfected the pancreatic cancer cells with negative control (NC) and MGAT1-overexpression (MGAT1-OE) constructs. Typically, the transfection process was carried out according to the manufacturer’s protocol within the recommended time frame. By using Lipofectamine 3000, we aimed to efficiently introduce the NC and MGAT1-OE RNA into the pancreatic cancer cells for subsequent analysis and to study the effects of gene overexpression on cellular processes and molecular pathways.

### 2.7 CCK-8 assay

We used the Cell Counting Kit-8 (CCK-8) assay to assess cell viability. Twenty-four hours after transfection, pancreatic cancer cells were seeded into 96-well plates at a density of 1500 cells per well in 200 μL of complete culture medium. The cells were then incubated at 37°C. For the CCK-8 assay, we added 10 μL of CCK-8 solution (Beyotime, Shanghai, China) to each well containing cells. After incubating at 37°C for 4 hours, the reagent reacted with the cells, producing a colorimetric change related to cell viability.

After the incubation period, we measured the optical density (OD450) using a microplate reader. The OD450 value reflects the absorbance of the formazan product generated by CCK-8, which is proportional to the metabolic activity and viability of the cells. By quantifying the OD450 values, we could evaluate the relative survival rate of the cells and compare different experimental conditions or treatment groups.

### 2.8 Transwell assay

The invasive capability of pancreatic cancer cells was assessed using the well-established Transwell assay. In this experiment, a specific number of pancreatic cancer cells (approximately 1 × 10^5) were seeded into specialized chambers. To evaluate invasive potential, chambers coated with Matrigel were used. The upper chamber contained serum-free medium to establish a chemotactic gradient, while the lower chamber contained complete DMEM medium to provide a favorable environment for cell movement. After 24 hours of incubation, cells that had successfully traversed the membrane were fixed with 4% paraformaldehyde solution. To visualize and quantify the invading cells, they were stained with 0.1% crystal violet. Stained cells were then observed and counted under a light microscope, allowing for the assessment of cell numbers and invasive capability.

### 2.9 Wound healing experiment

To evaluate the migratory ability of pancreatic cancer cells, we employed the wound healing assay. Transfected cells were cultured in six-well plates and incubated at 37°C until they reached approximately 80% confluence. A uniform wound was introduced into the cell monolayer using a sterile 200 μL pipette tip. After wounding, cells were rinsed twice with PBS to remove debris and then supplemented with serum-free medium. The migration of cells towards the wound area was recorded using an Olympus inverted microscope at 0 hours and 24 hours.

### 2.10 Statistical analysis

Statistical analyses were performed using R version 4.2.2 (64-bit) and its support packages. In addition, Python version 3.8 was used. Non-parametric Wilcoxon rank sum test was used to assess the relationship between two groups of continuous variables. Spearman correlation analysis was used to test the correlation coefficients. All statistical investigations were considered statistically significant at a significance level of P<0.05.

## 3 Results

### 3.1 Cell type identification and glycosylation scoring of PDAC single cell sequencing data

We obtained single-cell sequencing data of pancreatic ductal adenocarcinoma (PDAC) tumor tissue and adjacent normal tissue from the GSE197177 dataset for analysis. The tumor samples selected were primary, untreated PDAC tumor tissues. The Seurat software package was used for initial processing and analysis of the single-cell data. After reading the single-cell data from the tumor and normal groups, we created a SeuratObject containing 28,166 cells.

**Figure 1:**
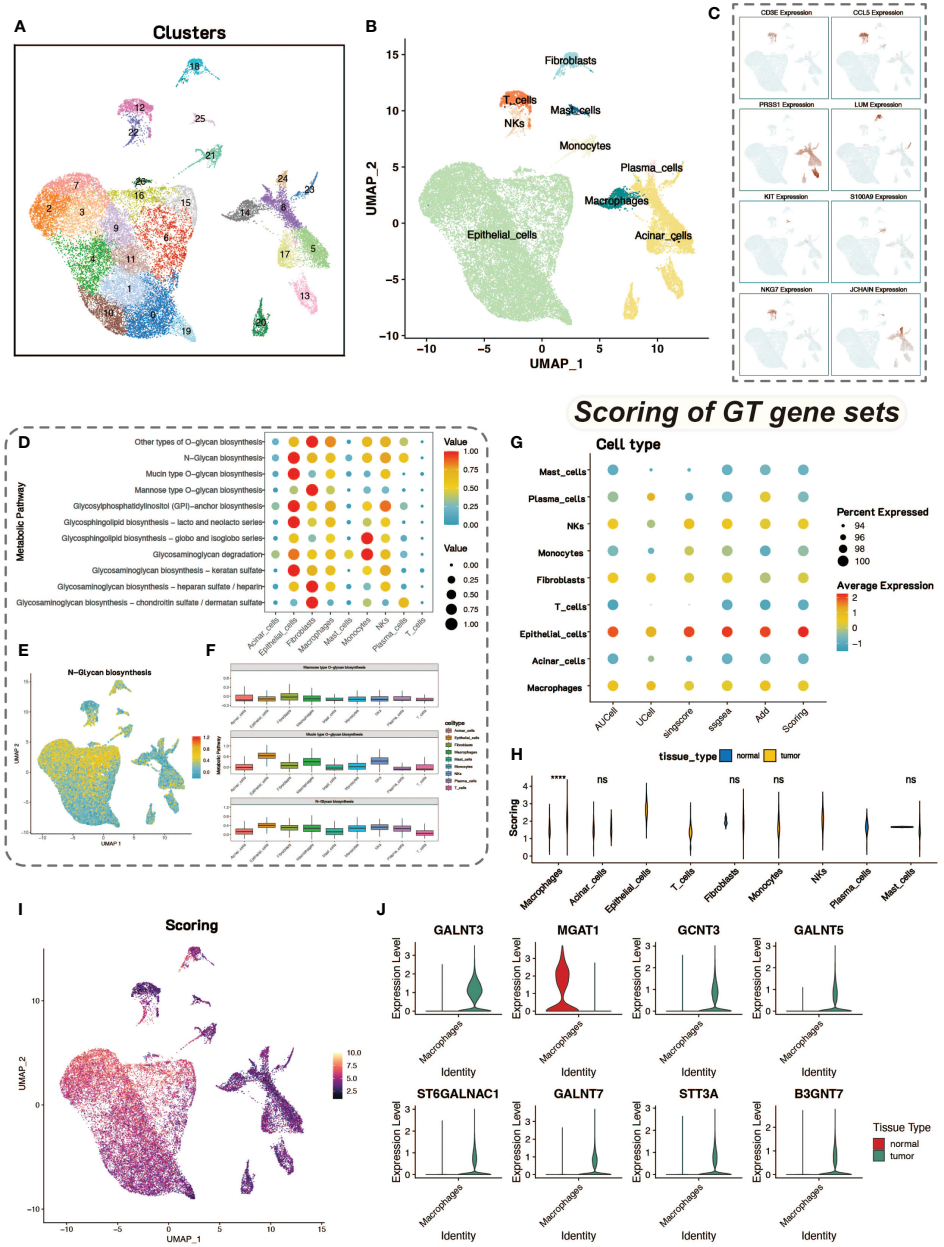
Dimensionality reduction, clustering and glycosylation scores. **(A)** UMAP visualization of single-cell data after dimensionality reduction and clustering, with colors representing different cell clusters, totaling 27 clusters. **(B)** UMAP plot of identified cell types, revealing 9 cell types. **(C)** Feature plot of 8 marker genes, showing their expression in different cell types. **(D)** Analysis of metabolic levels in various cell types within the tumor microenvironment. **(E)** UMAP display of N-Glycan biosynthesis metabolic scores in cells. **(F)** Box plot of glycosylation-related metabolic scores in different cell types. **(G)** Bubble plot of glycosyltransferase-related gene scores in various cell types using five different gene set scoring methods. **(H)** Violin plot showing the differences in scores between tumor and normal cells. **(I)** Heatmap of glycosyltransferase-related gene scores. **(J)** Violin plot of differentially expressed glycosyltransferase-related genes in macrophages between tumor and normal groups.

The preprocessing step was performed for quality control of the cells, and after normalization and identification of highly variable genes, downscaling and clustering were performed. After PCA downscaling, the top 10 principal components were selected for further analysis. MAP was used to visualize the clustering of the 27 cell clusters obtained (**Figure 1A**). Marker genes for pancreatic tissue cells were retrieved from the CellMarker database for cell type identification. After identifying the cell types, UMAP was again used to visualize the distribution and number of different cell types (**Figure 1B**). The expression of marker genes in different cell types was also demonstrated by UMAP (**Figure 1C**).

We conducted metabolic enrichment analysis of single-cell data using the scMetabolism package (**Figure 1D**). We focused on the glycosylation levels of various cell types, including N-linked and O-linked glycosylation (**Figures 1E, F**). To further explore the differences in glycosylation levels between tumor and normal tissues, we scored 185 glycosyltransferase-associated genes using five tools for calculating gene-set expression scores, including AUCell, UCell, singscore, GSVA, and addModuleScore. With these five gene-set scoring methods, the quantification of tumor and normal tissue glycosylation levels in different cell types in the tumor (**Figure 1G**). This combination of scoring methods also ensures the stability of the scoring results.

Statistical tests on the scoring results of tumor group cells and normal control group cells revealed that macrophages in the tumor group had significantly higher glycosyltransferase-related gene scores than those in the normal control group, with statistical significance (**Figure 1H**). Therefore, we focused on macrophages to study the impact of glycosylation on tumor cells and the tumor microenvironment. The results of glycosyltransferase-related gene scoring were also displayed on UMAP plots, showing consistency with the results from scMetabolism (**Figure 1I**). We conducted differential analysis between tumor group macrophages and normal group macrophages, identifying differentially expressed glycosyltransferase-related genes. These differentially expressed genes may play a significant role in influencing macrophage function and activity. The expression levels of the top eight genes were shown using violin plots (**Figure 1J**).

### 3.2 Macrophages with high and low glycosylation levels

**Figure 2:**
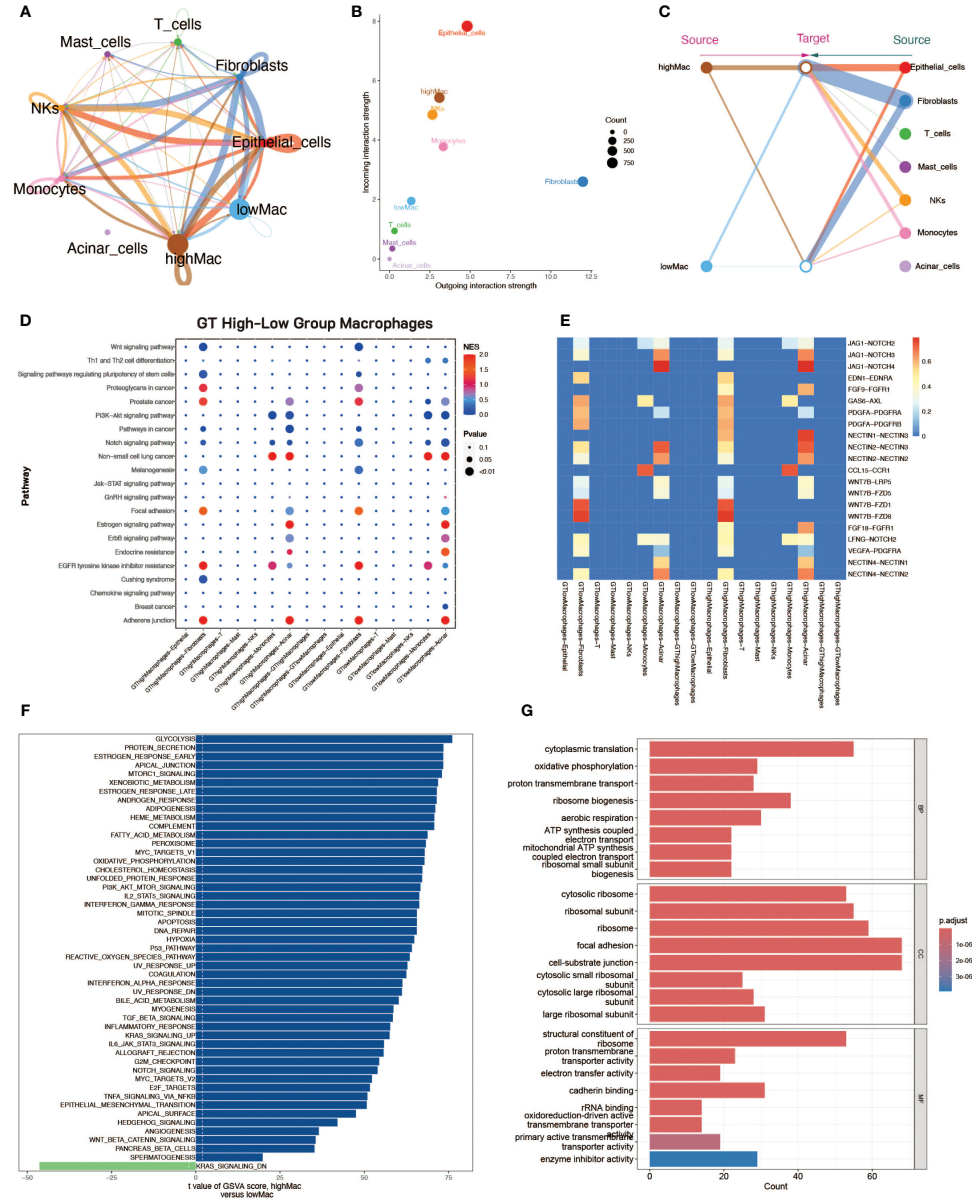
Cell communication analysis and enrichment analysis of macrophages with high and low glycosylation levels. **(A)** Cellular communication strength, demonstrating the strength of communication between different cell types, including highly glycosylated macrophages and lowly glycosylated macrophages. The thickness of the line represents the communication strength. **(B)** Scatter plot of communication, the horizontal axis represents the strength of emitting reciprocal signals and the vertical axis represents the strength of receiving reciprocal signals. **(C)** Diagram of communication structure with macrophages with high and low glycosylation levels on the left and other cell types on the right. **(D)** Scatter plot of signaling pathway strength. **(E)** Heatmap of signaling pathway constitutive ligand receptor intensities. **(F)** Scatter plot of the results of GSVA enrichment analysis, with blue bands on the right side for high glycosylation level macrophage enrichment pathway and green on the left side for low glycosylation level macrophage enrichment pathway. **(G)** Bar graph of GO enrichment analysis from both groups of macrophages at high and low glycosylation levels.

Macrophages in tumor tissues were classified into two groups of high and low levels (based on median scores) according to glycosylation scores. To explore the differences in the functional activities of macrophages at different glycosylation levels, we performed cell communication analysis and enrichment analysis. Cell communication analysis by CellChat showed that the communication strength of macrophages in the high-glycosylation level group was higher than that of the low-level group, and it was higher in both sending and receiving signals (**Figures 2A, B**). There were also differences in the communication patterns between the high and low levels of cells (**Figure 2C**). The results of CellCall analyses also demonstrated the differences in communication pathways between the high and low level groups (**Figures 2D, E**). GSVA enrichment analysis was performed on macrophages from both high and low level groups, and high glycosylation level macrophages were enriched for a large number of up-regulated pathways (**Figure 2F**). GO enrichment analysis was similarly used in macrophages from both high and low level groups (**Figure 2G**).

### 3.3 Processing and metabolic analysis of spatial transcriptomics data

**Figure 3:**
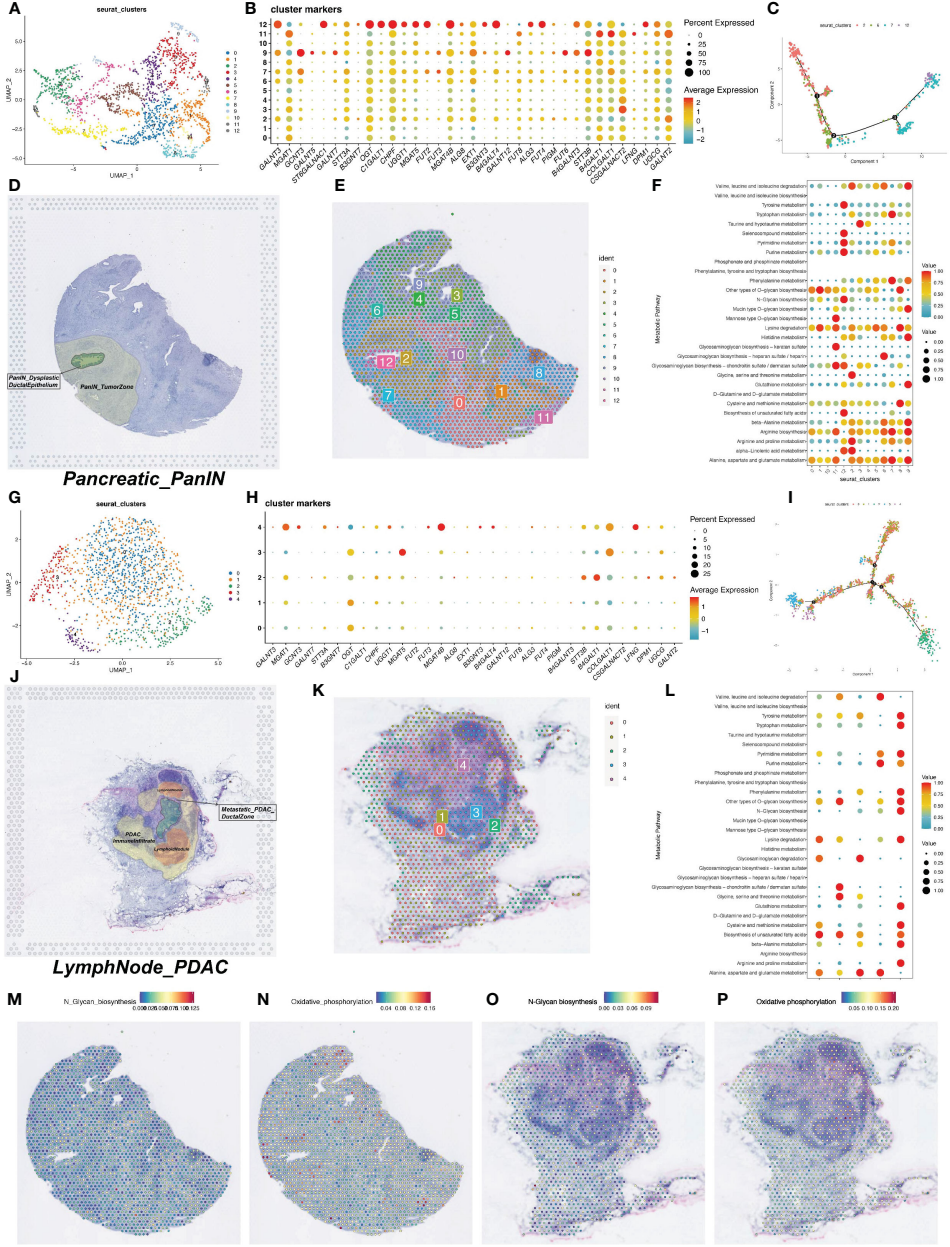
Spatial transcriptomics data on metabolic activity levels and glycosylation gene expression. **(A)** UMAP visualization of spatial transcriptome data after dimensionality reduction clustering to obtain 13 cell clusters. **(B)** Bubble plots of the expression of glycosylation-related genes in the spatial transcriptome data. **(C)** Trajectory plot of the proposed temporal analysis. **(D)** HE staining map of PDAC tumor tissue sections. **(E)** Display of the 13 cell clusters obtained by clustering in the section background. **(F)** Quantitative analysis of metabolic levels. **(G)** UMAP visualization of spatial transcriptome data after dimensionality reduction clustering to obtain 13 cell clusters. **(H)** Bubble plots of expression of glycosylation-related genes in spatial transcriptome data. **(I)** Trajectory plot of the proposed time series analysis. **(J)** HE staining map of PDAC tumor tissue sections. **(K)** Clustering obtained by presentation of 13 cell clusters in the background of sections. **(L)** Quantitative analysis of metabolic levels. **(M-P)** Heatmap of intensity of metabolic levels of N-terminal glycosylation and oxidative phosphorylation.

We downloaded two spatial transcriptomics datasets from the GEO database (GSE224422). One dataset was from a pancreatic intraepithelial neoplasia (PanIN) tissue (GSM7021871) and the other from a PDAC lymph node tissue (GSM7021872). After dimensionality reduction and clustering of the spatial transcriptomics data, we visualized the data using UMAP, resulting in 13 and 5 cell clusters for the two datasets, respectively (**Figures 3A, G**).

We first examined the expression of glycosyltransferase-related genes in the spatial transcriptomics data. In the PanIN dataset, glycosyltransferase-related genes showed significant expression in cell cluster 12 (**Figures 3B, H**). In the PanIN slice, cells in the ductal region and adjacent tissues mainly belonged to clusters 2, 6, 7, and 12. We performed pseudotime analysis on these four clusters to construct developmental trajectories. The pseudotime analysis showed distinct distribution ranges for the four clusters, with periductal tissue cells (cluster 2) appearing early in pseudotime and ductal epithelial cells (cluster 12) appearing at the latest stage (**Figure 3C**). The correlation between H&E-stained tissue slices and the spatial distribution of cell clusters helped us better understand the pseudotime analysis results. Pathology experts identified that the green area in the H&E-stained slice represented cancerous ductal epithelial cells, corresponding to cell cluster 12 (**Figure 3D**). Cell clusters 2 and 7 were the regions closest to the ductal epithelial cells and were most influenced by the cancerous cells (**Figure 3E**).

Pseudotime analysis was also conducted on the lymph node tissue slice, incorporating all cell clusters (**Figure 3I**). The results clearly showed that cell cluster 3 emerged at a later pseudotime stage, and comparative analysis with the H&E-stained slice indicated that cluster 3 represented the germinal center in the lymph node (**Figures 3J, K**). Metabolic enrichment analysis was performed on both tissue slices, identifying cell clusters with higher metabolic activity (**Figures 3F, L**). The N-Glycan biosynthesis and oxidative phosphorylation pathways were separately visualized on the tissue slice maps (**Figures 3M–P**).

### 3.4 Revealing developmental trajectories with spatial transcriptomics data

**Figure 4:**
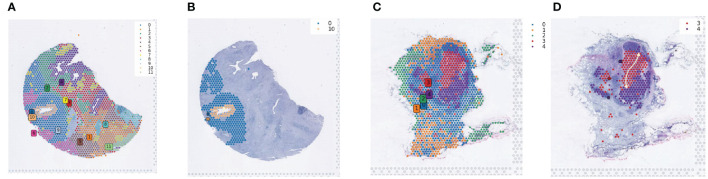
Spatial developmental trajectory analysis of PDAC tumor tissues. **(A)** Clustering of PDAC tumor section sequencing data using the louvain method in the stLearn software package; the clustering map shows the spatial distribution of different cell populations. **(B)** Spatial developmental trajectory maps of ductal epithelial cells and their surrounding cells in tumor tissue sections, drawn using the stLearn software package. **(C)** Clustering map of the second section data. **(D)** Developmental trajectory map of cells in and around the central region of lymph nodes.

Spatial transcriptomics data provide transcriptional information with precise cellular locations within tissues. We utilized the stLearn package for in-depth analysis of spatial transcriptomics data to explore tumor development processes, including invasion and metastasis. Using NumPy for data quality control and dimensionality reduction, and the Louvain method in stLearn for clustering, we identified 12 and 5 cell clusters in the pancreatic samples, respectively (**Figures 4A, C**). Focusing on cell clusters in the ductal regions of tissue slices, we reconstructed developmental trajectories using the Diffusion Pseudotime (DPT) algorithm and combined this with spatial coordinate information to reveal the progressive invasion and metastasis of tumor cells in pseudotime (**Figures 4B, D**).

### 3.5 Deconvolution and cell interaction analysis with combined spatial and single-cell data

Due to the resolution limitations of current spatial transcriptomics sequencing technologies, spatial transcriptomics data do not yet achieve the single-cell resolution of single-cell sequencing data. To address this limitation, we applied the SPOTlight deconvolution method, which infers the possible cell types and proportions at each location in the spatial transcriptomics data based on gene expression patterns of various cell types from pancreatic cancer single-cell sequencing data. This step allowed us to gain a deeper understanding of the spatial structure and function of tissues or cells, revealing interactions and communication between different cell types, as well as spatial heterogeneity and state changes.

**Figure 5:**
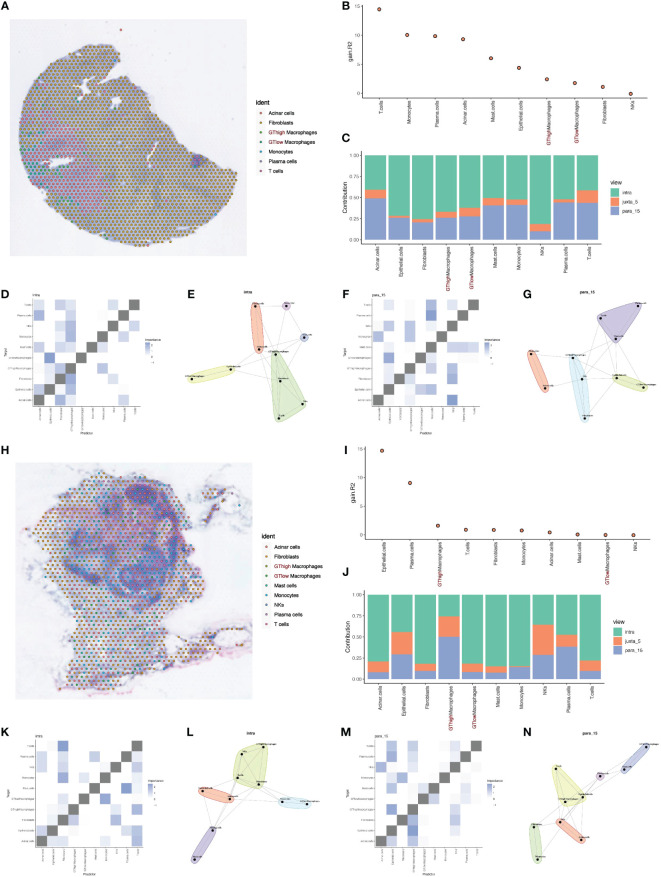
Deconvolution and cell interaction analysis based on spatial transcriptomics data. **(A)** Analysis of PDAC tumor tissue section data using the RCTD deconvolution method showing the spatial distribution probabilities of various cell types, including cells with high and low glycosylation levels. **(B)** Histogram of cell interactions intensity. **(C)** Bar graph showing the contribution of different views to the cell interactions assessed by the Mistyr software package, showing the relative importance of different views in cell interactions. **(D, E)** Heatmaps and network diagrams of cell interactions in the same view (intraview), revealing interaction strengths and patterns within the same cell type. **(F, G)** Heatmaps and network diagrams of cellular interactions in paraview15 view, showing interaction strengths and communication networks between cell types. **(H)** Results of RCTD inverse convolution analysis of the second layer of slice data showing the probability and spatial distribution of different cell types, including cells with high and low levels of mitochondrial autophagy. **(I)** Histogram of cell interactions intensity. **(J)** Bar graph showing the contribution of different views to PDAC tumor cell interactions, assessing the relative contribution of each view. **(K, L)** Heatmaps and network diagrams (intraview) of cellular interactions of the second layer of section data, showing the interaction relationships between the same cell types in the tumor environment. **(M, N)** Heatmaps and network diagrams of cell interactions in paraview15 views of the same tissue, revealing the interaction strengths and network structure of different cell types.

Additionally, we divided macrophages into high GT and low GT groups based on their median GT scores for more detailed deconvolution analysis. This analysis provided the spatial distribution and probabilities of various cell types on tissue slices (**Figures 5A, H**). Based on deconvolution analysis of the two tumor samples, we further applied the MISTy (Multiview Intercellular SpaTial modeling framework) framework for spatial transcriptomics cell interaction analysis. MISTy is an interpretable machine learning framework for analyzing single-cell, highly multiplexed, and spatially resolved data, offering insights into inter- and intra-cellular relationships.

Using MISTy, we could process a custom number of views, each describing different spatial contexts, such as intracellular regulation or paracrine regulation, and relationships between specific cell types. Our analysis results displayed the contribution of three different views to cell interactions through bar charts, revealing that intraview and paraview15 had the greatest contributions in the two tumor samples (**Figures 5B, C, I, J**). This highlighted the importance of intracellular regulation and paracrine regulation in tumor samples. Further heatmaps and network diagrams detailed the specific patterns of these two views in tumor samples, emphasizing significant interaction relationships between high and low GT macrophages and other cell types (**Figures 5D–G, K–N**).

### 3.6 MGAT1 gene in macrophages

**Figure 6:**
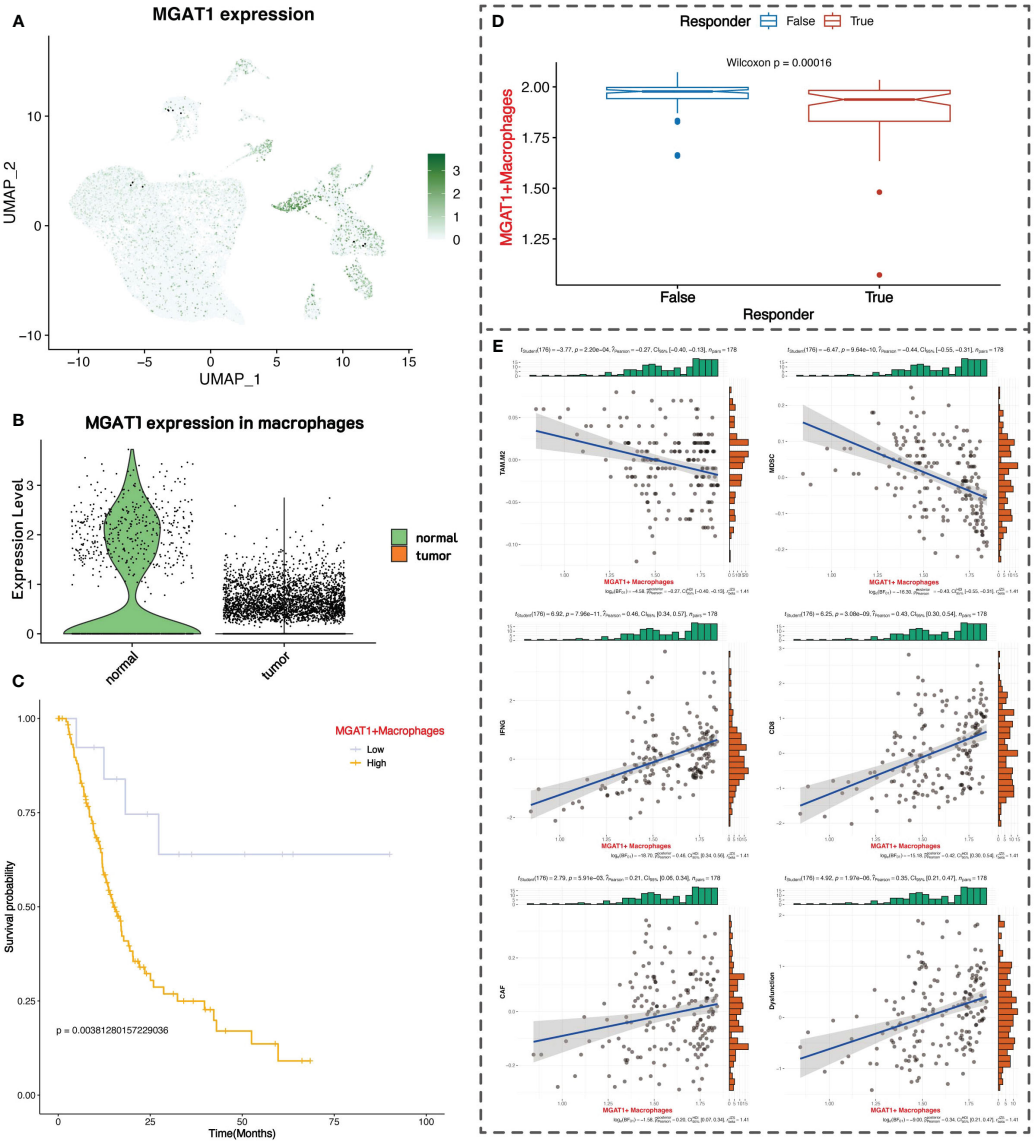
Clinical prognostic value of MGAT1. **(A)** UMAP plot of MGAT1 expression. **(B)** MGAT1 expression violin plot with macrophages in normal tissue on the left and macrophages in tumor tissue on the right. **(C)** K-M curve of overall survival of pancreatic cancer patients, with patients categorized into two groups of high and low expression based on the marker gene of MGAT1-positive macrophages. **(D)** Box plot of TIDE immunotherapy response. **(E)** Correlation of MGAT1-positive macrophages with immune-related markers.

We observed that the glycosyltransferase-related gene MGAT1 is highly expressed in macrophages in the normal group but is lowly expressed in the tumor group. MGAT1 encodes an enzyme that plays a crucial role in N-linked glycosylation. In the overall single-cell data, MGAT1 expression is primarily found in macrophages and acinar cells, with scattered expression in epithelial cells and other immune cells (**Figure 6A**). Specifically, MGAT1 expression in macrophages is significantly lower in the tumor group compared to the normal group (**Figure 6B**).

We downloaded RNA-seq data and survival information for pancreatic cancer from the Xena database, comprising 183 samples. We performed differential analysis between MGAT1-positive macrophages (MGAT1 expression > 0) and MGAT1-negative macrophages in the single-cell data to identify marker genes for MGAT1-positive macrophages. Using these marker genes, we conducted ssGSEA scoring on the RNA-seq data, dividing patients into high and low groups based on the median score for survival analysis. The results indicated that patients with low expression of MGAT1-positive macrophage marker genes had better prognoses, suggesting that lower MGAT1 expression in macrophages correlates with better tumor prognosis (**Figure 6C**).

Using the TIDE online tool for immunotherapy analysis, we found that patients with low expression of MGAT1-positive macrophage marker genes responded better to immunotherapy, further supporting the benefit of low MGAT1 expression in macrophages for prognosis (**Figure 6D**). We also mapped the correlation between the expression of MGAT1-positive macrophage marker genes and various immune cells and functions (**Figure 6E**).

### 3.7 Deconvolution analysis and spatial cell communication of MGAT1-related macrophages

**Figure 7:**
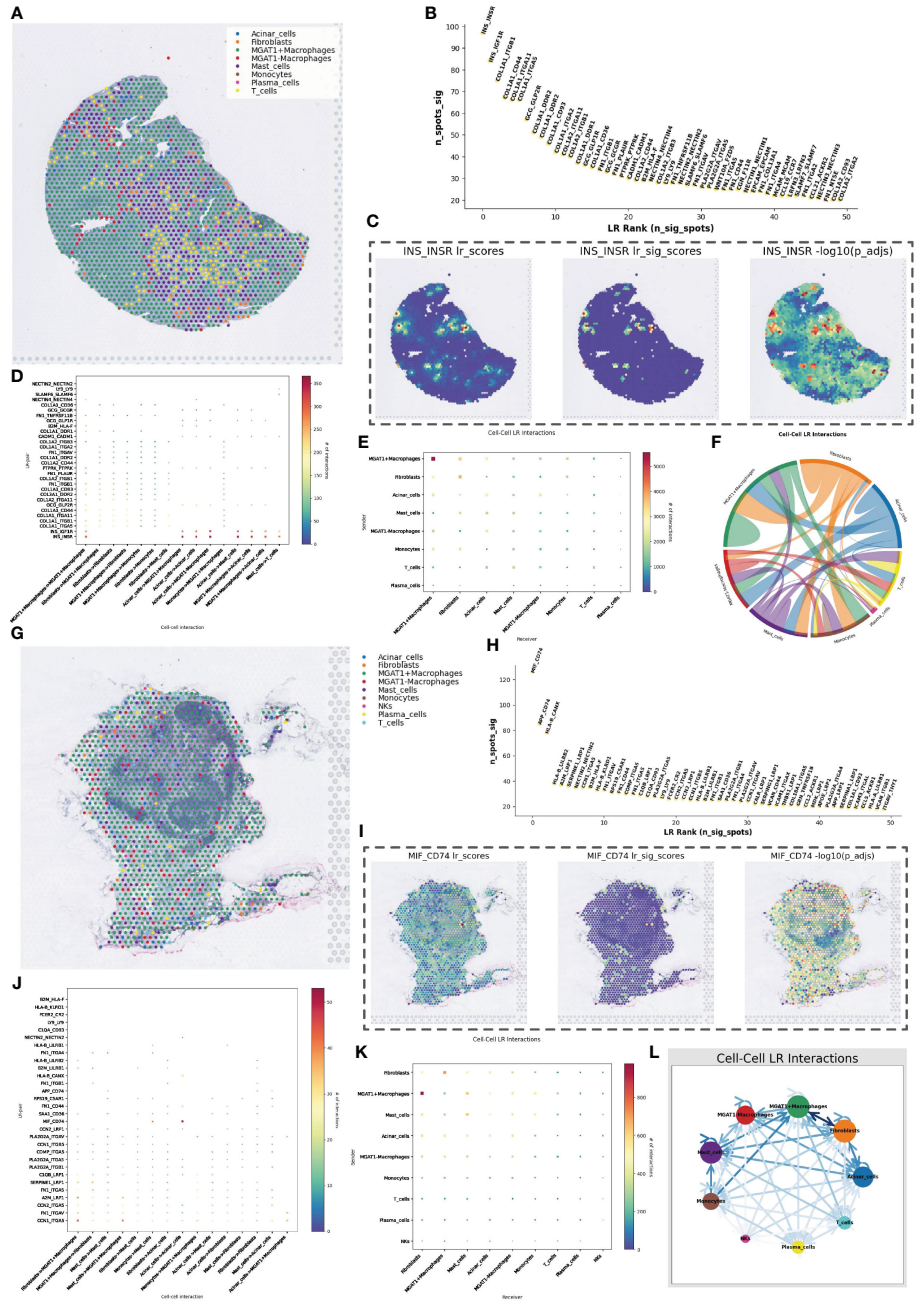
RCTD Inverse Convolutional Analysis and Spatial Cell Communication. **(A)** Cell type inference of spatial transcriptome data obtained from RCTD reverse convolution analysis, containing eight possible cell types, in which macrophages were classified into high and low glycosylation levels. **(B)** Spatial cellular communication inferred ligand receptor strength foldplot with significance in vertical coordinates and ligand receptor significance ranking in horizontal coordinates. **(C)** Heatmap of the intensity of hormone-mediated cellular communication mediated by pancreatic beta-cell secretion, with a heatmap showing scores and significance values, representing cells that maintain normal function. **(D)** Mate receptor activity in different types of cellular communication. **(E)** Heat map of cell communication strength between different cell types. **(F)** Cellular communication chord plot with each color representing a cell type. **(G)** Cell type inference of spatial transcriptome data obtained by RCTD reverse convolution analysis, containing nine possible cell types, in which macrophages were classified into high and low glycosylation levels. **(H)** Spatial cellular communication inferred from ligand receptor intensity fold plots. **(I)** Heatmap of MIF-CD74 ligand-receptor pair-mediated cellular communication intensity. **(J)** Ligand-receptor activity in different types of cellular communication. **(K)** Heatmap of cellular communication intensity between different cell types. **(L)** String diagram of cellular communication.

Based on MGAT1 expression in macrophages from single-cell data, we categorized macrophages into MGAT1-positive and MGAT1-negative groups. Using the gene expression patterns of each cell type from single-cell data, we performed deconvolution analysis on the spatial transcriptomics data using the RCTD method. This analysis provided the possible cell types at each spot in the spatial transcriptomics data (**Figures 7A, G**).

In the spatial cell communication of PanIN tissue slices, the INS_INSR and INS_IGF1R were the two most important ligand-receptor pairs. However, their significantly active regions were not near the ductal epithelial cells, i.e., not near the cancerous tissue (**Figures 7B, C**). Nevertheless, there were significant differences in cell communication mediated by these two ligand-receptor pairs between MGAT1-positive and MGAT1-negative macrophages (**Figure 7D**). The intensity of communication between cell types was visualized using heatmaps and chord diagrams (**Figures 7E, F**).

In the pancreatic cancer lymph node tissue, the MIF_CD74 ligand-receptor pair played an important communication role, primarily in the communication among acinar cells (**Figures 7H–J**). The communication intensity between cell types was also displayed using heatmaps and chord diagrams (**Figures 7K, L**).

### 3.8 Expression of MGAT1 in clinical subgroups

**Figure 8:**
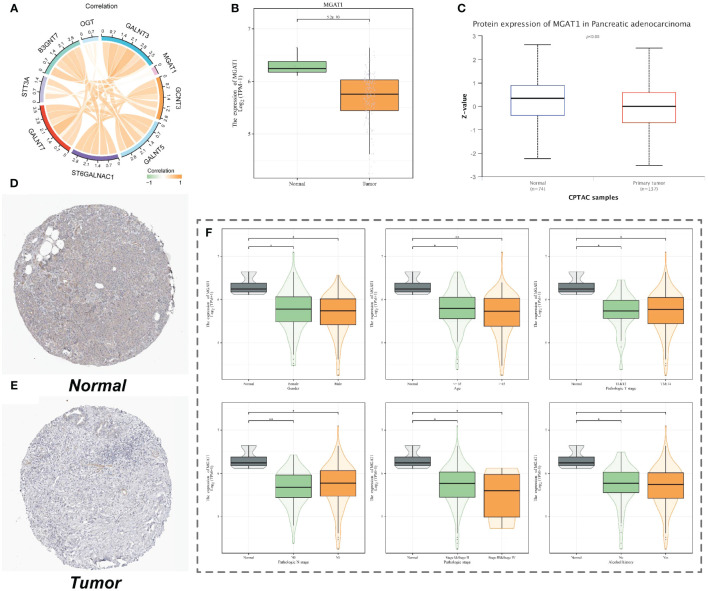
MGAT1 expression. **(A)** Correlation chord plot of the expression of nine important macrophage glycosylation-related genes. **(B)** Box plot of MGAT1 gene expression from TCGA pancreatic cancer data. **(C)** Box plot of MGAT1 protein expression. **(D)** Immunohistochemical staining images of MGAT1 in normal pancreatic tissues. **(E)** Immunohistochemical staining images of MGAT1 in pancreatic cancer tumor tissues. **(F)** Expression of MGAT1 in different clinical groups of patients.

We examined the expression correlation of MGAT1 and several other glycosyltransferase-related genes that were differentially expressed in macrophages using TCGA data. The results showed a weak correlation between MGAT1 and other glycosyltransferase-related genes (**Figure 8A**). In tumor tissues, MGAT1 expression was lower than in normal tissues, not only in macrophages but also in RNA-seq data (**Figure 8B**). More importantly, the protein expression level of MGAT1 was also lower in tumor tissues compared to normal tissues (**Figure 8C**). Immunohistochemistry results from HPA017432 staining of one pancreatic normal tissue and one pancreatic adenocarcinoma tissue showed lighter staining in the tumor group (**Figures 8D, E**). MGAT1 expression in clinical subgroups also revealed significant results (**Figure 8F**).

### 3.9 Association of MGAT1 with immune infiltration and prognosis

**Figure 9:**
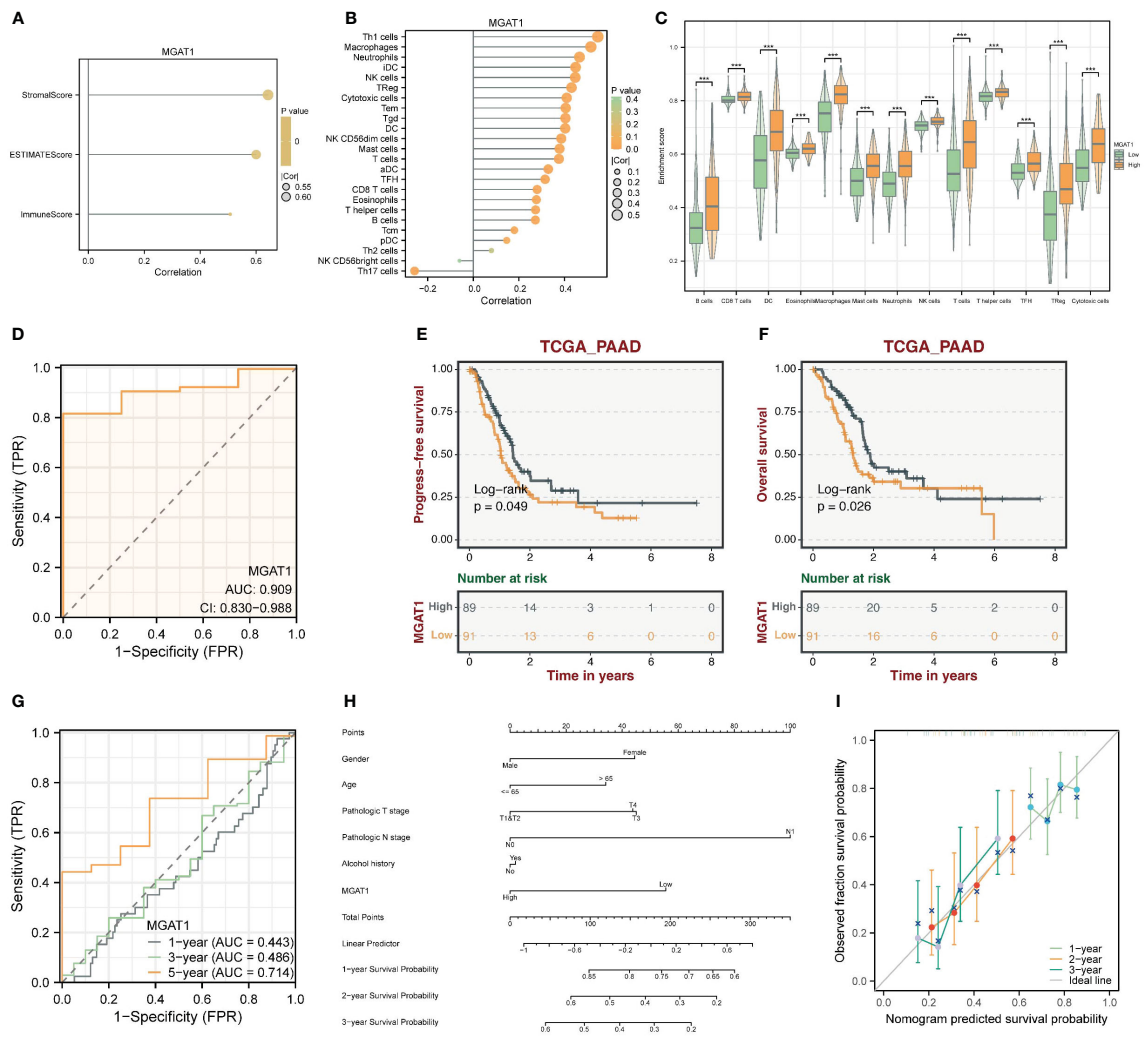
Clinically relevant studies of the MGAT1 gene. **(A)** Correlation between MGAT1 expression and stromal score and immunization score. **(B)** Correlation between MGAT1 expression and immune-infiltrating cells. **(C)** Box plots of immune infiltration in patients with high and low MGAT1 expression. **(D)** ROC curve of diagnostic potency of MGAT1 tumors. **(E)** K-M curves of MGAT1 expression groupings. **(F)** K-M curves of MGAT1 expression grouping. **(G)** ROC curves for prognostic modeling predicting 1-, 3- and 5-year survival probabilities. **(H)** Prognostic model nomogram composed of MGAT1 gene and clinically relevant shapes. **(I)** Prognostic model calibration curve.

The MGAT1 gene showed some correlation with the proportion of stromal cells in tumor samples (**Figure 9A**). Immune infiltration analysis indicated that patients with low MGAT1 expression had lower levels of immune infiltration compared to those with high MGAT1 expression. Given its correlation with stromal cells, we can infer that the prognostic benefit of low MGAT1 expression in pancreatic cancer patients is likely more related to its impact on stromal cells rather than immune cells (**Figures 9B, C**). Besides its prognostic impact, MGAT1 is also an important tumor marker gene, with an AUC of 0.909 from ROC testing (**Figure 9D**). The progression-free survival (PFS) and overall survival (OS) of patients with different MGAT1 expression levels were displayed using TCGA data, showing the highest five-year predictive accuracy with an AUC of 0.714 (**Figures 9E–G**). A prognostic nomogram incorporating MGAT1 and other clinical characteristics was constructed, and its accuracy was demonstrated using calibration curves (**Figures 9H, I**).

### 3.10 MGAT1 inhibits proliferation and migration of pancreatic cancer cells

**Figure 10:**
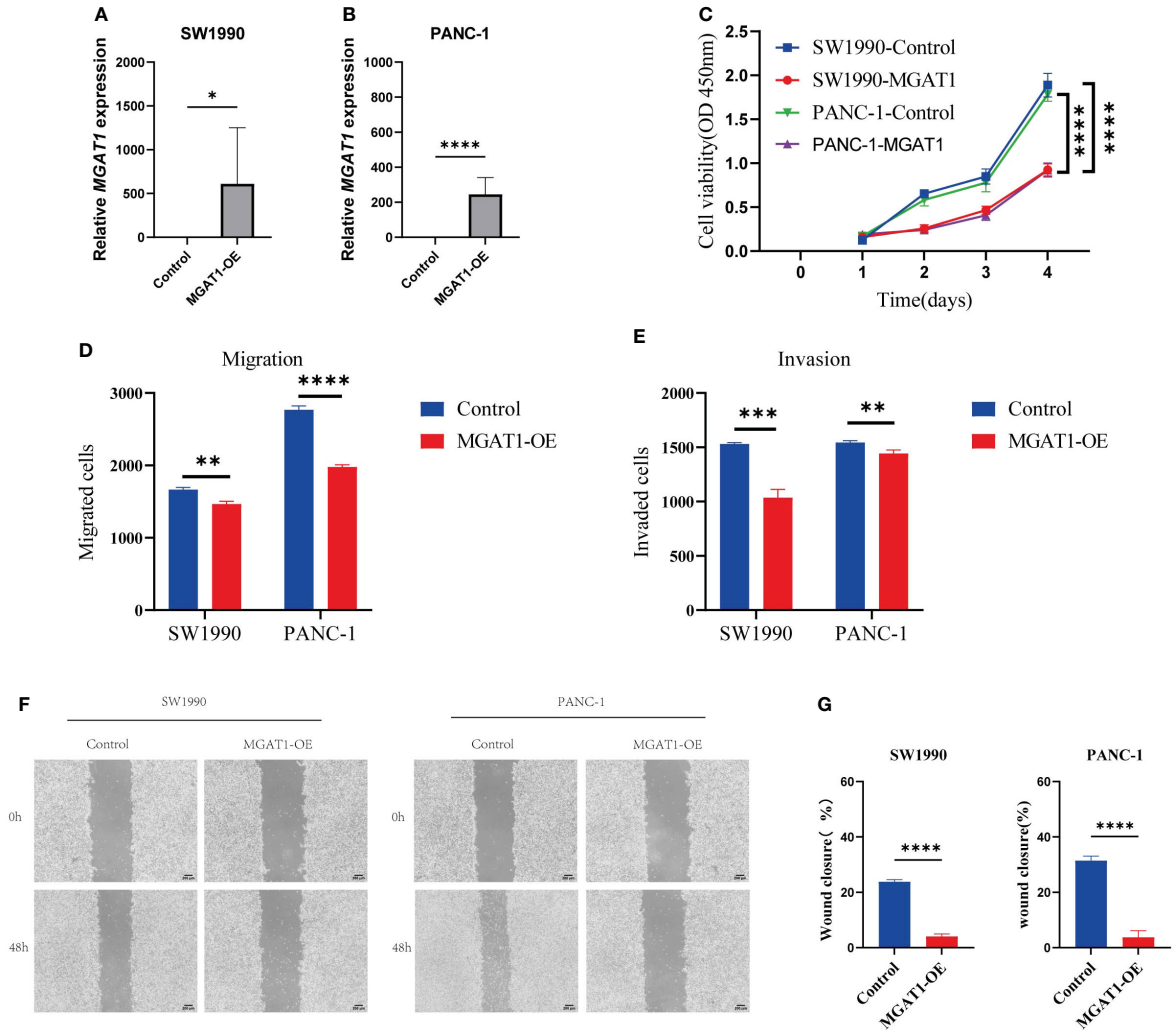
Cellular experiments. **(A)** Histogram of PCR analysis of MGAT1 expression in normal group versus MGAT1 overexpression group in SW1990 cell line. **(B)** Histogram of PCR analysis of MGAT1 expression in normal group versus MGAT1 overexpression group in PANC-1 cell line. **(C)** Line graph of the results of CCK-8 cell proliferation assay. **(D)** Histogram of cell migration assay results analysis. **(E)** Histogram of cell invasion assay results analysis. **(F)** Micrograph of cell scratch assay. **(G)** Histogram for analysis of cell scratch assay results. * indicates p < 0.05, ** indicates p < 0.01, *** indicates p < 0.001, **** indicates p < 0.0001, and ns indicates no statistical significance (p ≥ 0.05).

To investigate the potential role of MGAT1 in pancreatic cancer, we conducted *in vitro* experiments. First, we confirmed the overexpression of the MGAT1 gene using PCR (**Figures 10A, B**). We performed transfection experiments to overexpress MGAT1 in tumor cells and confirmed it using PCR. The CCK-8 assay showed that MGAT1 overexpression significantly inhibited cell proliferation (**Figure 10C**). The Transwell assay indicated that MGAT1 overexpression significantly reduced cell invasion and migration (**Figures 10D, E**). The wound healing assay also demonstrated that MGAT1 overexpression significantly inhibited cell migration (**Figures 10F, G**).

## 4 Discussion

PDAC is one of the most aggressive malignancies with a poor prognosis. Key characteristics of it include rapid disease progression and resistance to conventional therapies. The TME of PDAC is complex, comprising cancer cells, stromal cells, immune cells, and an extensive extracellular matrix. These components collectively promote tumor growth, metastasis, and therapeutic resistance (3, 18, 19).

Glycosylation, a post-translational modification process where glycans are enzymatically attached to proteins or lipids, plays a critical role in various biological processes, including cell signaling, immune response, and protein stability (20–23). Glycosyltransferases are crucial enzymes in the glycosylation process, adding glycans to proteins or lipids. Aberrant glycosylation is a hallmark of cancer, influencing tumor progression, metastasis, and immune evasion (24, 25). Dysregulation of glycosyltransferases in tumors can lead to the expression of unique glycan structures not present in normal tissues, affecting cell-cell interactions, signal transduction, and immune recognition. Therefore, the importance of glycosylation in tumor progression cannot be overlooked, especially the critical role of glycosyltransferases in regulating tumor cell behavior and immune evasion (7, 26, 27).

Our study found that the glycosylation levels in macrophages in the PDAC tumor group were significantly higher than in the normal group. This finding underscores the crucial role of glycosylation in the tumor microenvironment (28, 29). Using single-cell RNA sequencing and spatial transcriptomics data, we identified several important glycosylation-related genes and focused on the glycosylation patterns of macrophages. We observed the expression characteristics of these genes in different cell types, revealing the complexity of cell interactions within the tumor microenvironment. Notably, the expression of glycosyltransferase-related genes significantly impacted the function and activity of macrophages. Among these genes, MGAT1 was particularly important. Our research showed that the expression level of MGAT1 in macrophages was significantly correlated with PDAC prognosis. Aberrant expression of MGAT1 not only affected macrophage function and activity but also played a key role in cell communication and immune response (30). Overall, these findings provide new insights into the role of glycosylation in PDAC and offer crucial experimental evidence for MGAT1 as a potential therapeutic target. Future studies should further explore the role of MGAT1 in other cancer types and develop innovative therapeutic strategies targeting glycosylation pathways.

Our research indicates that glycosyltransferase-related genes play a significant role in the prognosis of PDAC. Specifically, the expression level of MGAT1 in macrophages was significantly correlated with patient prognosis. Combining TCGA data with survival analysis results, we found that patients with high MGAT1 expression had a significantly better prognosis than those with low MGAT1 expression. Our survival analysis showed that the survival rate of patients in the high MGAT1 expression group was significantly higher than that of the low MGAT1 expression group. This finding suggests that high MGAT1 expression may inhibit tumor progression and metastasis, leading to better prognosis. CCK-8 and Transwell assays demonstrated that overexpression of MGAT1 significantly inhibited the proliferation and migration of pancreatic cancer cells. Furthermore, wound healing assays showed that MGAT1 overexpression could significantly inhibit cell migration. We also explored the relationship between MGAT1 gene expression and immune therapy response. Therefore, the expression level of MGAT1 can serve as an important biomarker for PDAC prognosis and a potential indicator for evaluating the effectiveness of immune therapy (31, 32). In summary, high MGAT1 expression in PDAC may be a key factor leading to better prognosis. Future research should further explore the specific mechanisms of MGAT1 in tumor progression and develop targeted therapeutic strategies to improve the prognosis and treatment outcomes of PDAC patients.

Despite revealing the important role of MGAT1 in PDAC, our study has some limitations. First, the limited sample size and data heterogeneity may affect the generalizability and reliability of the results. Future studies should include larger sample sizes and integrate data from different sources for comprehensive analysis. Second, we primarily relied on single-cell RNA sequencing and spatial transcriptomics data, which, despite providing high-resolution information, also have limitations in sequencing depth and coverage. Future studies should combine other high-throughput technologies, such as mass spectrometry and multi-omics data, to fully elucidate the function of MGAT1. Third, our research focused on the impact of MGAT1 on macrophage function and tumor prognosis, with less emphasis on other glycosyltransferase genes. Future studies should expand the research scope to comprehensively understand the mechanisms of glycosylation in PDAC. Additionally, while our *in vitro* experimental data are relatively sufficient, *in vivo* experimental data are still lacking. Future research should validate the specific role of MGAT1 in PDAC using animal models. Finally, we explored the relationship between MGAT1 and immune therapy response, but the specific mechanisms remain unclear. Future studies should analyze how MGAT1 regulates the immune microenvironment and its application in immune therapy in depth.

## 5 Conclusion

This study investigates the role of glycosylation and its related genes in PDAC, particularly the glycosyltransferase MGAT1. Our results show that the glycosylation level of macrophages in the PDAC tumor group is significantly higher than that in the normal group. High expression of MGAT1 is associated with better patient prognosis, and its overexpression significantly inhibits the proliferation and migration of pancreatic cancer cells. This study provides important evidence for MGAT1 as a potential therapeutic target for pancreatic cancer, and we hope that future research will deepen these findings and promote the development and application of clinical treatment strategies.

## Data availability statement

The original contributions presented in the study are included in the article/supplementary material. Further inquiries can be directed to the corresponding authors.

## Ethics statement

Ethical approval was not required for the studies on humans in accordance with the local legislation and institutional requirements because only commercially available established cell lines were used.

## Author contributions

LaiJ: Writing – original draft, Visualization, Software, Formal analysis, Data curation, Conceptualization. LiuJ: Writing – original draft, Data curation, Conceptualization. SZ: Writing – original draft, Formal analysis, Data curation, Conceptualization. CJ: Writing – original draft, Visualization, Formal analysis, Data curation, Conceptualization. JH: Validation, Writing – original draft, Visualization, Formal analysis. HaiC: Writing – original draft, Visualization, Formal analysis. XZ: Writing – original draft, Visualization, Formal analysis. YF: Writing – original draft, Visualization, Formal analysis. ZY: Writing – original draft, Formal analysis. RW: Writing – original draft, Visualization, Formal analysis. GY: Writing – review & editing, Conceptualization. HaoC: Writing – review & editing, Conceptualization. BL: Writing – review & editing, Conceptualization.
